# Physical activity habits prevent psychological distress in female academic students: The multiple mediating role of physical and psychosocial parameters

**DOI:** 10.1016/j.heliyon.2024.e26626

**Published:** 2024-02-18

**Authors:** A. Levante, S. Quarta, M. Massaro, N. Calabriso, M.A. Carluccio, F. Damiano, F. Pollice, L. Siculella, F. Lecciso

**Affiliations:** a Department of Human and Social Sciences, University of Salento, Via di Valesio, 73100 Lecce, Italy; b Lab of Applied Psychology, Department of Human and Social Sciences, University of Salento, Via di Valesio, 73100 Lecce, Italy; c Department of Biological and Environmental Sciences and Technologies (DISTEBA), University of Salento, 73100 Lecce, Italy; d Institute of Clinical Physiology (IFC), National Research Council (CNR), 73100 Lecce, Italy

## Abstract

**Background:**

Psychological distress is recognised as the most common mental health difficulty in emerging adult (18-to-24-year-old) female academic students. This study aimed to test a novel model positing physical activity habits as a protective factor for psychological distress through the mediating role of physical and psychological parameters. Adherence to the Mediterranean diet and self-reported physical health status were included as physical parameters. Self-reported psychological health status and time spent on leisure activities were the psychological parameters considered.

**Method:**

Data were collected between April and May 2021. Correlation analyses and a multiple mediation model were computed on 411 online questionnaires filled out by 18-to-24-year-old female students from the University of blind (Italy).

**Results:**

The multiple indirect effects were significant (*β* = −0.088; *p* < 0.001). This means that physical activity habits reduce psychological distress through high adherence to the Mediterranean diet, a good self-assessment of one's physical and psychological health status, and more time spent on leisure activities outdoors, with friends, and with family members.

**Conclusions:**

Results show that academic policies should be adopted so as to design physical activity programmes that may improve the students’ healthy behaviours and social interactions, which, in turn, mitigate the detrimental effects of psychological distress.

## 1 Introduction

The transition from adolescence to adulthood may be stressful [1,2]. Emerging adulthood, spanning from ages 18 to 25, is characterised by identity formation, with young people developing as individuals and future employers/professionals [3,4]. This developmental stage is often marked by the beginning of university studies, which usually results in emerging adults having to deal with physiological and psychological changes [5], as well as an environment in which they have to face several challenges. Firstly, they need to learn how to manage an increased workload compared to high school [6]. Similarly, semester-long courses and exams mean academic students having to meet deadlines while keeping a balance between high standards of academic performance and social and family interactions [7]. Additionally, due to financial issues, a number of academic students need to work and study simultaneously, thus reducing the amount of time they devote to social interaction and leisure activities [2]. Taken together with individual predictors (e.g., age, gender), these challenges may contribute to the onset of mental health difficulties, in the form of inattention, fatigue, and psychological distress [8,9]. Evidence has shown that such mental health difficulties affect the physical [10] and psychosocial [11] well-being of academic students, even resulting in them performing worse and dropping out more frequently [12,13] than mentally healthier students.

The empirical literature on the university student population has revealed that psychological distress is recognised as the most common mental health difficulty in this population [[14], [15], [16], [17], [18]]. According to the Dual-Continua Model of Mental Health [19], psychological distress is the flip side of psychological well-being. In other words, psychological distress is a higher-order mental condition consisting of depressive symptoms (i.e., dysphoria, hopelessness, devaluation of life, self-deprecation), anxiety traits (i.e., situational anxiety, experience of anxious effects), and stress (i.e., chronic levels of non-specific arousal) [20,21].

The international empirical literature is consistent in highlighting the high prevalence of psychological distress in academic students and its detrimental effect on their well-being and academic performance. A study involving 10,000 academic students [22] has shown that more than 30% of them suffered from mental disorders (e.g., depression and anxiety). Furthermore, a meta-analysis by Regehr and colleagues [23], involving 1431 Western academic students, has reported that around half of the participants displayed moderate levels of psychological distress. In the US, the prevalence of psychological distress components (i.e., depression, anxiety, and stress) in academic students ranged from 33% to 40% [24]. As for the impact of psychological distress on well-being and academic performance, a study involving Australian academic students [25] has shown that the participants reported a higher level of psychological distress than the general population, with it being associated with lower achievement and underperformance.

The prevalence, severity, and effects of psychological distress in academic students [[26], [27], [28], [29], [30]] have highlighted the need to focus on this at-risk population, in terms of both research and public health. Therefore, a more in-depth examination of the factors contributing to psychological distress seems to be necessary.

Previous research has demonstrated the risk and predictive role of psychological distress in the academic student population for two sociodemographic variables, i.e., gender and age. More specifically, studies have shown that psychological distress, in the form of depression symptoms and anxiety traits, is one of the greatest challenges of modern times, especially for females [31] during emerging adulthood [32], when most of them are continuing with their education and/or beginning their professional career. As for gender vulnerability, Stallman [25] has found that female academic students reported higher levels of psychological distress than their male counterparts, data that are consistent with those observed in the general population [25]. Additionally, other studies [18,[33], [34], [35]] have shown that female academic students are more likely than their male counterparts to report reduced psychological well-being and be treated for mental health difficulties. In terms of the vulnerability to psychological distress due to the age stage, a study [25] has found that academic students aged 18 to 24 report the highest levels of psychological distress. Considering the paucity of studies solely focused on this target population and the growing interest in gender-specific research, this study aimed to expand knowledge of the relationship between the parameters analysed, paving the way for and/or improving gender- and age-specific training and/or intervention programmes that may prevent the detrimental effects of psychological distress on students’ well-being and academic performance. Therefore, a multiple mediation model was tested on Italian female academic students aged 18 to 24. More specifically, physical activity habits were posited as a protective factor against psychological distress through the multiple mediating roles of physical and psychological parameters. To date, no other studies focused on this target population seem to have been carried out. This study may expand knowledge of factors that may prevent psychological distress in this population in a developmental stage full of stressors.

In this paper, studies on the direct path between physical activity habits and psychological distress will be reviewed (Section 1.1). Furthermore, the empirical findings on the path between psychological distress and physical (Section 1.2) and psychological (Section 1.3) parameters will be summarised. Finally, due to the period (i.e., the 3-year COVID-19 pandemic) in which data were collected, an analysis will be carried out of the studies conducted during the COVID-19 lockdowns on all the parameters included in the multiple mediation model (Section 1.4).

### 1.1 Physical activity habits and psychological distress in academic students

Physical activity habits were posited as predictors of psychological distress. This parameter includes healthy habits that prevent diseases and harmful risk behaviours [36]. More specifically, according to Merino-Marban and Mayorga-Vega [37], physical activity habits encompass any body movement that leads to energy burning. Longitudinal [38] and intervention studies [[39], [40], [41]] have revealed a direct and negative path between physical activity habits and psychological distress in young adults. In other words, the more physical activity is done, the fewer mental health difficulties are experienced in terms of anxiety and depressive symptoms (as components of psychological distress). Previous research [42] has shown that physical activity may protect academic students’ well-being (i.e., the hedonic and eudaimonic dimensions of psychological well-being) through the mediating role of energy levels, leisure activities (i.e., time spent in nature), and quality social relationships.

Despite showing the benefits of physical activity habits, evidence [43,44] has also highlighted that the frequency of physical activity decreases in the shift from adolescence to adulthood. The university period is said to be critical [[45], [46], [47]], since academic students tend not to engage in sports due to academic and social demands and tasks [34], thus failing to meet the recommended minimum level of 150 min of weekly physical activity [48]. Similarly, evidence has demonstrated that academic students’ insufficient levels of physical activity may be associated with mental health difficulties [[49], [50], [51]]. Findings [[52], [53], [54]] have shown that female academic students tend to do less physical activity than their male counterparts, which results in them being vulnerable to psychological distress.

Physical activity habits are a protective factor not only for psychological distress, as they beneficially impact also other physical and psychological aspects. At a physical level, physical activity habits improve cardiorespiratory functions and body composition [[55], [56], [57], [58]], prevent diseases such as diabetes, osteoporosis, and stroke [44], reduce stress hormones [59], and are associated with high adherence to the Mediterranean diet [60]. At a psychological level, physical activity habits reduce morbidity in mental health difficulties, encourage social relationships [58,[61], [62], [63]], and contribute to developing individual values [55,56].

Considering the benefits brought by physical activity habits, this study aimed to test the simultaneous mediating role of two physical (i.e., adherence to the Mediterranean diet and self-reported physical health status) and psychological (i.e., self-reported psychological health status and time spent on leisure activities) parameters in the path between physical activity habits and psychological distress.

### 1.2 Physical parameters and psychological distress in academic students

Two physical parameters affecting psychological distress were considered, that is, adherence to the Mediterranean diet and self-reported physical health status. The first parameter, adherence to the Mediterranean diet, describes the healthiest dietary behaviour, due to its preventing physical and mental illnesses [[64], [65], [66]]. As it has been pointed out by numerous scholars [[67], [68], [69]], the Mediterranean lifestyle consists of dietary components and eating habits, as well as potentially protective factors including the conviviality of shared meals, daily activities at home or in the workplace, a regular sleep-wake routine, and meaningful social interactions (e.g., with friends and family members). The second physical parameter taken into account was self-reported physical health status, which refers to the individual's assessment of their own physical health condition.

Over the past decade, there has been an increasing interest [70,71] in the relationship between adherence to the Mediterranean diet and psychological distress in academic students. Several authors [[72], [73], [74]] have shown that adherence to the Mediterranean diet is associated with better mental health and low depressive symptoms (a component of psychological distress).

Some studies [70,[75], [76], [77], [78]] have demonstrated that the intake of healthy foods (e.g., fruit and vegetables) and regular healthy behaviours (e.g., having breakfast and the right number of meals every day) are associated with positive emotions, life satisfaction, and better academic performance. Conversely, nonadherence to dietary behaviours and consumption of unhealthy foods has been associated with mental health difficulties, and psychological distress in particular [74,79]. Findings have revealed that, in non-stressful situations, female academic students tend to adhere to the Mediterranean diet more than their male counterparts [52,[80], [81], [82]]. On the other hand, in stressful times, female academic students are more likely than males to increase their consumption of unhealthy foods (e.g., sugary and fatty foods) [81].

Existing evidence has demonstrated a strong relationship between self-reported physical health status and psychological distress in academic students [83,84]. In other words, the higher the students’ perception of their own physical health status, the lower the perceived psychological distress. This may support the idea that perceiving oneself as being physically healthy may act as a buffer against psychological distress. A study by Gitonga Rintaugu and Ngetich [85] has revealed that female academic students have a lower perception of their physical health status than their male counterparts.

### 1.3 Psychological parameters and psychological distress in academic students

The two psychological parameters affecting psychological distress that were included in the mediation model were self-reported psychological health status and time spent on leisure activities. For the specific purposes of this study, self-reported psychological health status was considered to consist of three components. The first component is the hedonic one [86,87], which results from feelings of happiness, optimism, and satisfaction. The second component is the eudaimonic one [88,89]in the form of the meaningfulness of one's life, self-actualization, and self-efficacy. The third component is the social one [19], which consists in the individual's ability to succeed in their own social interactions. For the specific purposes of this study, the psychological parameter “time spent on leisure activities” was conceived of as social initiative. That is the frequency with which people engage in leisure activities that encourage social interactions (i.e., with friends and/or family members) and/or are done in specific environments (e.g., nature) in which social interactions are fostered.

As for the path between self-reported psychological health status and psychological distress, a recent systematic review [90] on the factors contributing to the mental health of academic students has revealed that psychological strength and mental well-being are positively associated. This means that academic students who report positive mental states, optimism, more frequent social interactions and more friendships are more likely to experience fewer mental health difficulties. Therefore, a positive state of mind may act as a buffer against psychological distress in academic students who appear vulnerable during the transition to emerging adulthood. In addition, a review by Campbell and colleagues [90] has highlighted that female academic students report low levels of mental health and a low assessment of their psychological health status.

As for the association between leisure activities and psychological distress, an outdated study by Ragheb and Mckinney [91] has revealed that the beneficial effects of participation in leisure activities reduce academic students’ stress levels. More recent findings [42] have shown a positive impact of leisure-time physical activity done in nature on any mental health indices, although the study in question does not provide any details about differences across genders. Nevertheless, a study involving female adults [92] has revealed a direct and positive association between engagement in leisure activities and general mental health.

### 1.4 Impact of the COVID-19 pandemic on psychological distress in Italian academic students

The COVID-19 pandemic, which broke out in 2020, was caused by the Sars-CoV-2 virus. As it is well known, in order to prevent the spread of the infection, drastic emergency measures were imposed by governments worldwide. Such measures included lockdowns, social distancing, the use of masks, and the closing of any kind of shop and recreational facility, including gyms. Furthermore, schools and universities shifted from face-to-face to distance learning. Although these restrictive measures were effective in containing the spread of the infection [[93], [94], [95], [96]], they negatively impacted the mental health of the general population [[97], [98], [99], [100]]. People showed high levels of depressive symptoms, anxiety, concern, and psychological distress [[101], [102], [103], [104], [105]]. In addition, lockdowns led people to change their behaviour and routines, also in terms of physical activity, eating habits, sunlight exposure, and social interactions [97,[106], [107], [108], [109], [110], [111], [112], [113], [114], [115]]. This resulted in studies being carried out on at-risk populations such as that of academic students.

Although home-based physical activity in the form of online workout sessions, aerobic training, rope skipping, and dancing was recommended during lockdowns [[116], [117], [118]], a systematic review by Lopez-Valenciano and colleagues [119] has reported that during the first year of the pandemic (i.e., 2020), academic students reduced both their light/mild-intensity and high/vigorous-intensity physical activity. Further studies carried out during the pandemic revealed that academic students’ reduced levels of physical activity were associated with an increase in sedentary activities [42,[120], [121], [122], [123], [124], [125]].

Studies involving Danish, Greek, and French academic students [[126], [127], [128]] have shown that a growing percentage of participants experienced psychological distress during the COVID-19 lockdowns. Research on the Italian academic student population [42] has found that more than 50% of participants reported mild to extremely severe levels of depression and stress (as components of psychological distress), while ∼40% of the recruited students showed mild to extremely severe levels of anxiety (as components of psychological distress). Both a study by Petersen and colleagues [129] involving Danish academic students and research carried out by Talapko and colleagues [130] on Croatian academic students have revealed that students dramatically reduced their physical activity, which resulted in high levels of psychological distress, in terms of depression symptoms and stress.

As for the physical parameters included in the sequential mediation model (i.e., adherence to the Mediterranean diet and self-reported physical health status), a study [131] comparing Mediterranean and non-Mediterranean academic students has highlighted the underconsumption of healthy foods and low adherence to the Mediterranean diet during lockdowns. Similarly, a systematic review [132] on the general adult population has reported that studies involving academic students showed a reduction in the adherence to the Mediterranean diet. As for physical health status, evidence [133] has demonstrated that academic students reported several physical problems (e.g., weight gain, body aches), which affected their perception of their own physical health condition during the pandemic.

In terms of the psychological parameters included in the sequential mediation model (i.e., self-reported psychological health status and leisure activities), studies carried out during the pandemic have shown that academic students perceived their life as being disrupted by the pandemic [134] and reported feelings of dissatisfaction [135]. Leisure activities were not permitted during the first months of the pandemic. However, a study [136] has revealed that even when Covid-19 emergency measures became less harsh, academic students were reluctant to engage in leisure activities for fear of contagion, withdrawing socially.

Therefore, during the pandemic, all of the existing relationships hypothesised in the multiple mediation model were confirmed. Nevertheless, no previous studies tested them using a multiple mediation model. This paper aimed to extend knowledge of such relationships, focusing on the age- and gender-specific at-risk population of Italian female academic students. Two hypotheses were formulated.

HP1 *Physical activity habits are expected to negatively predict psychological distress through the multiple mediating positive roles of two physical parameters (i.e., adherence to the Mediterranean diet and self-reported physical health status*).

HP2 *Physical activity habits are expected to negatively predict psychological distress through the multiple mediating positive roles of psychosocial parameters (i.e., self-reported psychological health status and time spent on leisure activities*).

**Fig. 1:**
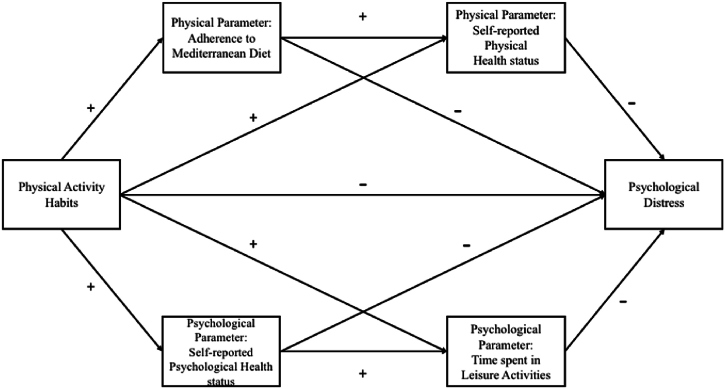
Hypothesised multiple mediation model.

Fig. 1 shows the hypothesised multiple mediation model, directions and paths.

## 2 Materials and methods

### 2.1 Ethical Statement and study design

This study was designed as a research project of the University of Salento in order to identify the physical and psychological factors affecting the psychological distress of female students. Due to the population involved in the study (academic students from the University where the research group is based), the research protocol was first submitted to the Data Protection Officer for approval. On the privacy matter, the Data Protection Office is required to not ask for the birth year of the student, but the age class. The study was designed and conducted in compliance with the Italian rules and regulations governing research practices in psychology, the ethical requirements for research in the field of psychology, and the General Data Protection Regulation (GDPR). Two previous studies [42,72] were conducted that were mainly focused on physical parameters, including physical activity, adherence to the Mediterranean diet, and outdoor leisure time. Consistent with the aforementioned evidence, two inclusion criteria were used in this study to extract the sub-sample, i.e. [1], female students [2] aged 18 to 24.

This study was conducted in accordance with the ethical standards of the Declaration of Helsinki and was approved by the Research Ethics Committee of the Department of Human and Social Sciences of the University of Salento (no. 0056300).

An online questionnaire, a link to which is available upon request, was created using Google Forms and was circulated via a mailing list at the University of Salento. A presentation of the research project was attached to the email students received. Data were collected cross-sectionally between April and May 2021. The online questionnaire took 15–20 min to complete. Before filling out the online questionnaire, participants were informed about the aims of the study and their rights via an information sheet. Participants signed an e-consent both to participate to the research and for the publication of all their anonymized data. Furthermore, they were also informed that they could exit the online questionnaire at any time. No compensation for participation was offered.

### 2.2 Participants

Non-probability sampling was used, with 411 female academic students voluntarily participating in the study. The age class of the sample is 18–24 years. Further information on the sample was collected. Most (n = 378) of the participants were disease-free. The majority (n = 343) of the participants did not have a partner. Students were classified according to the BMI categories defined by the World Health Organization (available online at: https://www.who.int/westernpacific/health-topics/obesity), with 11.6% of participants being underweight (<18.5 kg/m^2^), 72.6% having a normal weight (18.5–24.9 kg/m^2^), 11.6% being overweight (25.0–29.9 kg/m^2^), and 4.1%. being obese (≥30 kg/m^2^). Finally, the respondents’ family income was clustered: it was extremely low for 6.3% (<500€/month) of the participants, low for 15% (500-1000€/month), medium-low for 29.5% (1000–1500€/month), medium-high for 22.7% (1500–2000€/month), high for 17.5% (2000–2500€/month), and extremely high for 9.1% (>2500€/month).

### 2.3 Measures

*Psychological Distress*. In order to assess psychological distress levels, the 21-item short version of the Depression Anxiety Stress Scales (DASS-21) [20] was used (e.g., *“I had extreme difficulty in starting what I had to do”; “I feel discouraged and depressed”*). The rating scale was as follows: 0 = “Did not apply to me at all”; 1 = “Applied to me to some degree, or some of the time”; 2 = “Applied to me to a considerable degree, or a good part of the time”; and 3 = “Applied to me very much, or most of the time”. Distress was calculated as the average of all items. The higher the score, the higher the distress levels. Evidence [20,137] has shown that the measure has good psychometric properties, in terms of structural validity, internal consistency, temporal stability, and criterion-oriented validity.

*Physical Activity Habits*. In order to assess physical activity habits, an item developed for the specific purposes of this research project and used in a previous study [42] was chosen (i.e., “*In the last months, how often have you done physical activity?”*). Response options included 0 = “Never”; 1 = “Occasionally, but not regularly”; 2 = “Regularly, less than 150 min per week”; 3 = “Regularly, 150 min or more per week”. The cut-off points were: low (never or occasionally) and moderate/high (regularly).

#### 2.3.1 Physical parameters

*Adherence to the Mediterranean Diet.* Adherence to the Mediterranean diet was assessed using the 14-MEDAS questionnaire previously validated for use with the Italian sample population [138,139]. The questionnaire consisted of 14 questions about the main food groups consumed as part of the Mediterranean diet, with each question being assigned a score of 0 or 1 (in line with the recommendations for adherence to the Mediterranean diet). Overall, the score ranged from 0 to 14. Higher scores indicated a higher adherence to the Mediterranean diet. Previous studies [138,140] have validated the measure for use with Mediterranean and non-Mediterranean cultures.

*Self-Reported Physical Health Status.* The subscale of the WHOQoL-Bref questionnaire [141] was used to assess the participants' physical health status (i.e., “*Are you totally satisfied with your physical status?”*). Response options ranged from 1 (“Not at all”) to 5 (“Extremely”). The higher the score, the higher the students’ satisfaction with their own physical health status. Evidence [141] has shown good internal consistency, criterion and concurrent validity, and test-retest reliability.

#### 2.3.2 Psychological parameters

*Time Spent on Leisure Activities*. In order to assess the amount of time students spent on leisure activities, three items were developed. Participants were asked how much time they spent carrying out leisure activities in nature (“*In the last month, how often have you spent time in nature?”*), with family members (“*In the last month, how often have you spent time with your family?”*), and with friends (“*In the last month, how often have you spent time with your friends?”*). Response options included: 1 = “Never”; 2 = “Rarely”; 3 = “Sometimes”; 4 = “Often”; and 5 = “Always”. The score was computed as the average of the three items. The higher the score, the higher the time spent on leisure activities. Previous studies [42,72] have validated the measure.

*Self-Reported Psychological Health Status*. The subscale of the WHOQoL-Bref questionnaire [141] was used to assess the participants' psychological health status (i.e., “*Are you totally satisfied with yourself?”*). Response options ranged from 1 = “Not at all” to 5 = “Extremely”. The higher the score, the higher the students’ satisfaction with their own psychological health status. Evidence [141] has shown good internal consistency, criterion and concurrent validity, and test-retest reliability.

### 2.4 Statistical Plan

Statistical analyses were computed using the Statistical Package for the Social Sciences (SPSS) Version 25.0. Due to all the questions being made mandatory, no imputation methods were used to handle missing data. The Kolmogorov-Smirnov test (K–S test) was used to test whether the distribution of scores of each considered variables (i.e., physical activity habits, psychological distress, adherence to the Mediterranean diet, self-reported psychological and physical health status) significantly differed from normal distribution. Pearson's *r* bivariate *c*orrelation analysis was carried out to examine the associations between variables, i.e., physical activity habits, psychological distress, adherence to the Mediterranean diet, self-reported psychological and physical health status. BMI and family income were also included as control variables in the correlation analysis. PROCESS macro for SPSS was used to test the multiple mediation model [142]. Model 82 by Hayes was used in PROCESS macro to test the mediating role of multiple mediators, that is, adherence to the Mediterranean diet (M1), psychical (M2) and psychological (M3) health status, and time spent on leisure activities (M4), in the relationship between physical activity habits (x; independent variable) and psychological distress (y; dependent variable). Five-thousand bootstrap samples and a 95% bias-corrected confidence interval (95% CI) were used to examine the significance of the multiple mediation effect [142]. The statistical significance level was set at *p* < .05.

## 3 Results

### 3.1 Preliminary and Descriptive results

The K–S test was not significant for all scores, that is, physical activity habits (K–S = 0.214; p > 0.050), psychological distress (K–S = 0.097; p > 0.050), adherence to the Mediterranean diet (K–S = 0.118; p > 0.050), self-reported physical (K–S = 0.323; p > 0.050) and psychological (K–S = 0.244; p > 0.050) health status, and time spent on leisure activities (K–S = 0.104; p > 0.050). This means that the distribution of the scores was not significantly different from normal distribution.

Table 1 shows the mean and standard deviation of each considered parameter.

**Table 1:** Mean and standard deviation of each considered parameter.

	Mean (Standard Deviation)	Range
Physical activity habits	1.30(1.07)	0–3
Psychological distress	1.04(0.54)	0–3
Adherence to the Mediterranean diet	6.40(1.71)	0–10
Self-reported physical health status	2.75(0.85)	0–4
Self-reported psychological health status	2.26(0.98)	0–4
Time spent on leisure activities	2.04(0.68)	0–4

### 3.2 Correlation analyses

Pearson correlation matrices showing the relationship between measures are listed in Table 2. BMI was associated positively with psychological distress (*r* = 0.188; *p* = 0.0131) and negatively with self-reported physical (*r* = −0.341; *p* < 0.001) and psychological (*r* = −0.104; *p* < 0.001) health status. Correlation analyses showed that family income was positively associated with physical activity habits (*r* = 0.164; *p* = 0.001) and self-reported psychological health status (*r* = 0.134; *p* = 0.005). A negative association between family income and psychological distress (*r* = −0.174; *p* < 0.001) was found. Physical activity habits were correlated negatively with psychological distress (*r* = −0.118; *p* < 0.001) and positively with adherence to the Mediterranean diet (*r* = 0.230; *p* < 0.001), self-reported physical (*r* = 0.397; *p* < 0.001) and psychological (*r* = 0.161; *p* < 0.001) health status, and time spent on leisure activities (*r* = 0.191; *p* < 0.001).

**Table 2:** Correlations between variables.

	[1]	[2]	[3]	[4]	[5]	[6]	[7]
BMI *n* = 411	−0.030	−0.138**	0.118*	0.005	−0.341***	−0.104*	−0.050
Family income [1] *n* = 411		0.164**	−0.174***	0.076	0.158	0.134**	0.078
Physical activity habits [2] *n* = 411			−0.118***	0.230***	0.397***	0.161***	0.191***
Psychological distress [3] *n* = 411				−0.108***	−0.332***	−0.693***	−0.258***
Adherence to the Mediterranean diet [4] *n* = 411					0.229***	0.166***	0.064*
Self-reported physical health status [5] *n* = 411						0.428***	0.252***
Self-reported psychological health status [6] *n* = 411							0.285***
Time spent on leisure activities [7] *n* = 411							–

Note: *p < 0.05, **; *p* < 0.10; ***p < .01.

Furthermore, both physical parameters, i.e., adherence to the Mediterranean diet (*r* = −0.108; *p* < 0.001) and self-reported physical (*r* = −0.332; *p* < 0.001) health status, were negatively associated with psychological distress. Similarly, both psychological parameters, i.e., self-reported psychological health status (*r* = −0.693; *p* < 0.001) and time spent on leisure activities, (*r* = −0.258; *p* < 0.001) were negatively associated with psychological distress.

### 3.3 Multiple mediation model

**Fig. 2:**
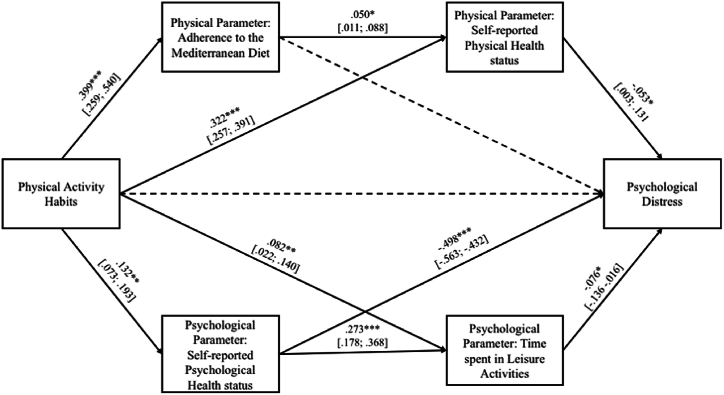
Results of the multiple mediation model. The model was significant [*F*_(1,439)_ = 13.488, *p* < 0.001]. The total (*β* = −0.088; *p* < 0.001) effect of female academic students' physical activity habits on their psychological distress was significant. The direct path was not significant (*β* = 0.006; *p* > 0.050).

The results of the regression analyses are shown in Fig. 2.

The path between physical activity habits and adherence to the Mediterranean diet (*β* = 0.399; *p* < 0.001) and that (*β* = 0.322; *p* < 0.001) between the predictor and the students’ self-reported physical health status were both significant. In addition, findings revealed a positive effect of adherence to the Mediterranean diet on self-reported physical health status (*β* = 0.050; *p* = 0.024) and a negative path between self-reported physical health status and psychological distress (*β* = −0.053; *p* = 0.050). Finally, the path between adherence to the Mediterranean diet and psychological distress was not significant.

The analysis of psychological parameters showed that the path between physical activity habits and self-reported psychological health status (*β* = 0.132; *p* < 0.001) and that between the predictor and time spent on leisure activities (*β* = 0.082; *p* = 0.005) were both significant. Furthermore, results showed a positive effect of self-reported psychological health status on time spent on leisure activities (*β* = 0.273; *p* < 0.001) and a negative path between the latter and psychological distress (*β* = −0.076; *p* = 0.012). Finally, the path between self-reported psychological health status and psychological distress was significant (*β* = −0.498; *p* < 0.001).

In terms of indirect effects, only the mediating role played by adherence to the Mediterranean diet in the path between physical activity habits and psychological distress was not significant (*β* = 0.000; BootLLCI = −0.020; BootULCI = 0.018). The mediating effect of physical health status on the relationship between physical activity habits and psychological distress was significant (*β* = 0.034; BootLLCI = 0.001; BootULCI = 0.072). The third significant indirect effect concerned the multiple mediation effect of both physical parameters (i.e., adherence to the Mediterranean diet and self-reported physical health status) on the path between physical activity habits and psychological distress (*β* = −0.002; BootLLCI = 0.000; BootULCI = 0.006). In other words, physical activity habits negatively affect psychological distress through the mediation of high adherence to the Mediterranean diet and high perception of physical health status.

The indirect effects of the multiple mediation of the psychological parameters were all significant. More specifically, psychological health status mediated the path between physical activity habits and psychological distress (*β* = 0.130; BootLLCI = −0.190; BootULCI = −0.072). Time spent on leisure activities significantly mediated the relationship between the predictor and the outcome (*β* = 0.012; BootLLCI = −0.028; BootULCI = −0.001). Finally, the multiple mediation effect of both psychological parameters (i.e., psychological health status and time spent on leisure activities) in the path between physical activity habits and psychological distress was significant (*β* = 0.005; BootLLCI = −0.011; BootULCI = −0.011). This means that physical activity habits negatively affect psychological distress through the mediation of high perception of psychological health status and more time spent on leisure activities (in nature, with family members, and friends).

Overall, the findings confirmed the hypotheses formulated in this study.

## 4 Discussion

This study is situated within existing research into the significant role that physical activity may have in reducing mental difficulties, and especially psychological distress, in emerging adults. Although a review by Paluska and Schwenk [143] has highlighted that no conclusive evidence has been found that physical activity prevents the onset of mental difficulties, physical activity may diminish the likelihood of developing mental disorders. Consequently, investigating the role of multiple and simultaneous factors impacting this relationship becomes crucial in terms of research and clinical issues. No previous studies seem to have been carried out on the matter. Therefore, this paper may significantly contribute to both the understanding of the link between physical activity and mental health and the identification of protective factors.

The impact of physical activity habits on psychological distress was tested through the mediating role of physical and psychological parameters in the vulnerable population of female academic students. As female academic students aged 18 to 24 have been demonstrated to be vulnerable to psychological distress [18,25,[33], [34], [35]]this study focused on this at-risk population in order to expand knowledge of each of the relationships considered and open up new fields of research on the factor(s) that may act as a buffer against psychological distress in this developmental stage full of stressors. It is worth noting that results should be interpreted in light of the challenging and stressful period (i.e., the COVID-19 pandemic) in which data were collected. Nevertheless, preliminary results showed that the recruited female academic students reported low levels of both psychological distress and physical activity. The scores for adherence to the Mediterranean diet, self-reported physical and psychological health status, and time spent on leisure activities were average. Overall, preliminary results showed no critical scores for any of the considered parameters.

Additionally, the associations between the considered parameters and sociodemographic variables (i.e., BMI and family income) confirmed the findings of previous studies. More specifically, a heavier weight, in terms of BMI, leads people (i.e., students) to adhere to physical activity habits [144] and perceive their physical [145] and psychological [146] health status more negatively. Furthermore, findings highlighted that better economic status, in terms of family income, results in higher levels of physical activity [147,148], a better perception of one's psychological health status, and a decrease in psychological distress [149]. Due to these being correlation analyses, a more in-depth investigation of the causal interplay between these variables should be carried out in future studies involving both female academic students and other populations. Moreover, further studies comparing high- and low-income countries should be designed in order to understand whether cultural factors might affect the interplay between the considered parameters and/or vary across cultures.

Correlation analyses confirmed both previous evidence and the expected relationships in the mediation model. Indeed, physical activity habits are associated with all the considered parameters. More specifically, low psychological distress correlated with a high frequency of physical activity [150], high adherence to the Mediterranean diet [151], better perception of one's own physical/psychological health status, and longer time spent on leisure activities. Based on such promising associations, the interplay was tested cross-sectionally using a more complex mediation model.

Despite the cross-sectional nature of the study, findings confirmed the hypotheses formulated, thus encouraging further reflections on the topic. To begin with, results confirmed that physical activity habits may be a buffer against psychological distress in female academic students, through high adherence to the Mediterranean diet, a good self-assessment of the students’ physical and psychological health status, and more time spent on leisure activities in nature, with friends, and with family members.

The paths and direct/indirect effects analysed lead to further considerations. Firstly, no direct path between physical activity habits and psychological distress was found, in contrast with what has been revealed by studies carried out during [129,130] and before [[52], [53], [54]] the Covid-19 pandemic. It is worth noting that during the COVID-19 pandemic physical activity habits were negatively affected by emergency measures. Indeed, in the first months of the pandemic, physical activity was not permitted, whereas less restrictive measures were later introduced, with access to facilities being staggered and people having to maintain social distancing and wear a face mask. Although these aspects may have negatively affected the frequency of physical activity, Italian female academic students perceived them as a minor source of psychological distress compared to other factors, such as fear of contagion and/or financial difficulties [152,153]. Due to the period in which data were collected, it would be interesting to investigate and expand knowledge of this direct path in the at-risk population in question in ordinary conditions, in the absence of emergency- and/or health-related stressors.

Despite the lack of a direct path, findings highlighted the indirect effects of physical and psychological parameters in reducing female academic students’ psychological distress. In line with the mastery hypothesis [154] as a psychological mechanism affecting the relationship between physical activity and psychological distress, those who do physical activity may adopt other strategies to continue improving their mental health. Although this psychological theoretical model requires more in-depth investigation, the physical and psychological parameters here hypothesised as mediators may become additional resources for individuals to reduce mental difficulties.

Results partially confirmed HP1 in terms of the role played by physical parameters (i.e., adherence to the Mediterranean diet and self-reported physical health status). However, findings showed that adherence to the Mediterranean diet did not act as a mediator in the path between physical activity habits and psychological distress when not associated with other parameters. In other words, adherence to healthy eating habits (i.e., adherence to the Mediterranean diet) may act as a buffer against psychological distress when female academic students also perceive themselves as being physically healthy. As it has been pointed out by other scholars [67,155], a Mediterranean lifestyle can play a protective role because it implies both eating habits and other protective factors, such as personal involvement in food preparation, conviviality, adequate hydration, rest and relaxation, and shared meals, which may improve one's self-perception of their physical health status. This was further confirmed by the direct and positive path between adherence to the Mediterranean diet and self-reported physical health status. To sum up, these preliminary results may support the consumption of fruit, vegetables, unprocessed cereals, olive oil, nuts, and seafood, with low/moderate consumption of poultry and red meat, based on the principles of the Mediterranean diet, as a factor to improve self-perception of one's physical health status, which, in turn, may reduce psychological distress.

Findings also showed the protective role of the psychological parameters mediating the path between physical activity habits and psychological distress. Consistent with HP2, a high frequency of physical activity was found to foster a positive state of mind, in terms of optimism, self-actualization, and satisfaction in social interactions. Furthermore, getting fit was shown to encourage the involvement of female academic students in more leisure activities, positively affecting their mental difficulties (i.e., psychological distress).

Based on the above-mentioned results, the potential of physical activity in improving healthy/social behaviours and self-perception in female academic students may be outlined. The novel mediation model tested in this study provides an overview of the simultaneous processes that physical activity sets in motion to prevent and/or decrease psychological distress. Findings may suggest that female academic students who are inclined to get fit may also be willing to adopt healthy eating habits, such as cooking their own food, reducing the consumption of (sweet) junk food, and/or avoiding eating unhealthy foods [156]. If they get the appropriate levels of nutrients, they feel better physically, being more energetic and less tired/fatigued. Simultaneously, physical activity habits also impact psychological health status, as they may promote a positive state of mind, make students feel happy, efficient, socially skilled, and ready to engage in leisure activities in nature and/or with friends and/or family members.

The analysis of such findings results in further research questions that should be investigated in future studies. Could the path directions be related to the historical and stressful conditions (i.e., the COVID-19 pandemic) in which data were collected or could they be related to gender-specific issues? Due to male academic students being more inclined than their female counterparts to do physical activity [52,157,158], could the path directions be confirmed? These and other questions could be formulated and/or research could be carried out to explore and expand knowledge of this topic. Further studies retesting the multiple mediation model are required to inform academic services and policies.

Results should be interpreted in light of some limitations. The first limitation concerns the characteristics of the sample (i.e., gender, age, and cultural setting). Although choosing the specific sample in question may have limited the applicability and generalisability of the findings, the aim of this study was a more in-depth investigation of the vulnerable population in question. Future studies may focus on people in other developmental stages, in order to find out whether the direction and the magnitude of the interplay may be confirmed and/or to explore the role of sociodemographic variables in multiple interactions. Due to the restrictions imposed by the Data Protection Office of the University, the study cannot explored the impact of students’ age (in the form of continuous variable) on the considered psychological dimensions. Further studies should investigate this issue more in details.

Although results cannot be generalised to non-Mediterranean countries, they are in line with previous studies [71,155], which have reported a strong impact of the Mediterranean diet on psychological well-being.

A second limitation is related to the fact that data were collected only from a single university in southern Italy. Although this may affect the generalisability of the results, it may also encourage further studies in other universities both in Italy and abroad, so as to understand the interplay between cultures. Due to the cross-sectional nature of the study, inverse or reciprocal relationships cannot be excluded. Further longitudinal studies are required to better investigate findings and validate the tested multiple mediation model. In addition, data should be interpreted carefully, as they were gathered during the 3-year COVID-19 pandemic in Italy. Therefore, as findings provide a picture of a specific and challenging period, the model should be replicated in ordinary circumstances, so as to confirm the relationships identified and increase its generalisability. Finally, in order to expand knowledge and understand whether the considered relationships may be affected by the field and year of study a student is in, future research should take into account these academic parameters.

Overall, the results of this study confirmed previous evidence [42,72] on the key role played by physical activity in the well-being of the academic population. The new issues addressed by this study concern (a) expanding knowledge of the protective role played by physical activity habits in encouraging academic students to adopt healthy eating habits and increase social interaction, thus reducing mental health difficulties; and (b) testing the interplay between physical and psychological parameters in a subgroup of the academic population, i.e., female students. Furthermore, the two main strengths of this paper should be highlighted. Firstly, this study tested a new way to examine the interplay between physical and psychological parameters in mitigating the psychological distress in a vulnerable population. Secondly, results provided interesting knowledge of the protective role played by non-psychological factors that could be used in the treatment of mental difficulties.

Findings also provided several directions and inputs for the development of new academic policies and services. To begin with, due to the beneficial impact of physical activity on the well-being of female academic students, academic associations may design and implement gender-specific physical activity programmes, with exercise to be done indoors and/or outdoors. These programmes could foster social interaction between academic students, thus reducing their psychological distress. In addition, in order to promote healthy eating habits based on the principles of the Mediterranean diet, academic policies may be developed to encourage the cafes on campus to offer healthier foods, such as seasonal vegetables and fruit, and fewer unhealthy snacks. Similarly, healthier meals should be introduced in university canteens. Finally, counsellors could design gender-specific and person-centred interventions focused on the assessment of female students’ self-perceived physical and psychological health status, in order to prevent the development of a negative self-image and promote coping strategies.

## Availability

Data associated with the current study have not been deposited into a publicly available repository. Data will be made available on request.

## Informed consent

Participants signed an e-consent both to participate to the research and for the publication of all their anonymized data.

## Funding

none.

## CRediT authorship contribution statement

**A. Levante:** Writing – review & editing, Writing – original draft, Software, Methodology, Investigation, Formal analysis, Data curation, Conceptualization. **S. Quarta:** Writing – review & editing, Methodology, Investigation. **M. Massaro:** Writing – review & editing, Methodology, Investigation, Conceptualization. **N. Calabriso:** Investigation. **M.A. Carluccio:** Investigation. **F. Damiano:** Methodology, Investigation. **F. Pollice:** Investigation. **L. Siculella:** Writing – review & editing, Methodology, Investigation, Conceptualization. **F. Lecciso:** Writing – review & editing, Supervision, Methodology, Investigation, Conceptualization.

## Declaration of competing interest

The authors declare that they have no known competing financial interests or personal relationships that could have appeared to influence the work reported in this paper.
